# Identification and pathogenicity of *Fusarium* species associated with wilting and crown rot in almond (*Prunus dulcis*)

**DOI:** 10.1038/s41598-024-56350-5

**Published:** 2024-03-08

**Authors:** Ana López-Moral, Begoña Isabel Antón-Domínguez, María Lovera, Octavio Arquero, Antonio Trapero, Carlos Agustí-Brisach

**Affiliations:** 1 Departamento de Agronomía (Unit of Excellence ‘María de Maeztu’ 2020-2024), ETSIAM, Universidad de Córdoba, Campus de Rabanales, Edif. C4, 14071 Córdoba, Spain; 2 Departamento de Fruticultura Mediterránea, IFAPA, Alameda del Obispo, 14004 Córdoba, Spain

**Keywords:** Microbiology, Plant sciences

## Abstract

Severe *Fusarium* wilt and crown root symptoms were observed in almond orchards in Portugal. The present study elucidates the etiology of the disease through molecular, phenotypic, and pathogenic characterization. Three *Fusarium* isolates from Portugal were tested and 12 *Fusarium* isolates from almond from Spain were included for comparative purposes. Their identity was inferred by phylogenetic analysis combining *tef*1 and *rpb*2 sequences. The Portuguese isolates were identified as *Fusarium oxysporum sensu stricto* (*s.s.*), and the Spanish isolates as *Fusarium nirenbergiae*, *F. oxysporum* (*s.s.*), *Fusarium proliferatum*, *Fusarium redolens* (*s.s.*), *Fusarium sambucinum* (*s.s.*), and *Fusarium* sp. Fungal colonies and conidia were characterized on potato dextrose agar (PDA) and on Synthetischer Nährstoffarmer agar, respectively. The colonies had a variable morphology and their color ranged from white to pale violet. Typical *Fusarium* micro- and macroconidia were characterized. Temperature effect on mycelial growth was evaluated on PDA from 5 to 35 °C, with optimal growth temperature ranging between 16.8 and 26.4 °C. The pathogenicity of *F. oxysporum* was demonstrated by inoculating almond plants (‘Lauranne’) grafted on GF-677 or Rootpac 20 rootstocks. A significant reduction in plant growth, wilting, and xylem discoloration was observed, with Rootpac 20 being more susceptible than GF-677. Infections were also reproduced using naturally infested soils. Almond plants (‘Lauranne’) were inoculated with isolates of all *Fusarium* species, with *F. redolens* from Spain and *F. oxysporum* from Portugal being the most aggressive.

Almond [*Prunus dulcis* (Mill.) D.A. Webb] decline has been described as emerging complex disease in the main growing regions of this tree nut worldwide. Affected trees show wilting and leaf necrosis, gum production, general decline, and occasionally plant death. Wood discoloration, sectorial necrosis, and xylem discoloration are the typical internal symptoms of the disease^1–4^. This syndrome has been associated with a wide diversity of fungal trunk pathogens including species that belong to *Botryosphaeriaceae*, *Diaporthaceae*, *Diatrypaceae*, *Calosphaeriaceae*, *Cytosporaceae*, *Pleurostomataceae* or *Togniniaceae* families. They have been observed causing different almond syndromes that have been called as Botryosphaeriaceae canker, Ceratocystis canker, Cytospora canker, Diaporthe canker, Collophorina canker, Eutypa or Pallidophorina canker^3^. Recently, the association of all these pathogens causing general decline in almond has been renamed as almond decline syndrome (ADS)^1^.

In addition to all these fungal trunk pathogens associated with ADS, soilborne pathogens belonging to *Nectriaceae*, such as *Fusarium* species, have also been reported causing wilting, crown rot, stem canker and gumming in almond trees^1,5^. Although *Fusarium* genus comprises a broad diversity of species and strains with saprophytic behaviour in the soil, certain special forms or strains of *Fusarium* spp. are pathogenic and they are able to cause a negative impact in many crops^6^. Indeed, *Fusarium* spp. have been considered pathogens of economically important perennial crops such as avocado^7^, banana^8^, cashew^9^, citrus^10^, grapevine^11,12^, mango, papaya and pineapple^13^ and nut crops^2,14–17^. In these woody crops, *Fusarium* spp. are associated with wilting, cankers, root rot and plant death. Regarding the association of *Fusarium* species with *Prunus* spp. damages, Moreno et al.^18^ reported the susceptibility of almond to *Fusarium euwallaceae* infections, causing dieback and vascular streaking. *Fusarium euwallaceae* is a vascular pathogen vectored by *Euwallacea* sp. nr. *fornicatus*, the ambrosia beetle polyphagous shot hole borer (PSHB), and it has been described as the causal agent of *Fusarium* dieback on several hardwood tree species^14^. Recently, *F. euwallaceae* has been recovered from almond, nectarine (*Prunus persica*) and apricot (*P. armeniaca*) trees showing vascular streaking also associated with colonies of *E. fornicatus* in South Africa^19^. Chehri et al.^20^ evaluated the almond susceptibility to seven *Fusarium* species (*Fusarium anthophilum*, *F. chlamydosporum*, *F. eumartii*, *F. graminearum*, *F. longipes*, *F. nygamai*, *F. oxysporum*, *F. proliferatum*, *F. scirpi* and *F. solani*) recovered from the rhizosphere soil of forest trees, with *F. eumartii*, *F. oxysporum* and *F. solani* resulting in the most aggressive species causing stem rot in almond plants. *Fusarium oxysporum* has also been reported causing root and crown rot on sweet cherry (*P. avium*) in British Columbia (Canada). The affected trees showed gumming from leaf scar and crown areas in self-rooted trees, and the disease progress to leave wilt until the trees collapsed in summer^21^. In addition, Markakis et al.^5^ reported a severe stem canker syndrome in almond trees caused by *F. solani* in Greece.

Recently, several *Fusarium* species belonging to *Fusarium fujikuroi, F. oxysporum, F. redolens* and* F. sambucinum* species complexes have been isolated together with fungal trunk pathogens associated with ADS in southern Spain^1^. However, the pathogenicity of these *Fusarium* species to almond has not been yet demonstrated. In addition, severe symptoms of *Fusarium* crown root, vascular streaking and gumming were observed in a commercial almond orchard in southern Portugal. To date, the role of *Fusarium* species in almond infections associated with canker diseases and tree decline is still uncertain. Thus, the main goal of this study was to elucidate the etiology of a specific syndrome of *Fusarium* crown root, vascular streaking and gumming on almond in southern Portugal. To this end, *F. oxysporum* strains collected from a commercial almond orchard from southern Portugal showing severe attacks of wilting and crown root were characterized molecularly and phenotypically, and the pathogenicity was tested under several environmental conditions in almonds plants of cv. Lauranne grafted on GF-677 or Rootpac 20 (R20) rootstocks. In addition, *Fusarium* spp. isolates previously associated with the decline of almond trees in Spain by Antón-Domínguez et al.^1^ were included in this study to test their pathogenicity and to compare their aggressiveness with those from almond tree in Portugal.

## Results

### Field surveys and fungal isolation

**Figure 1:**
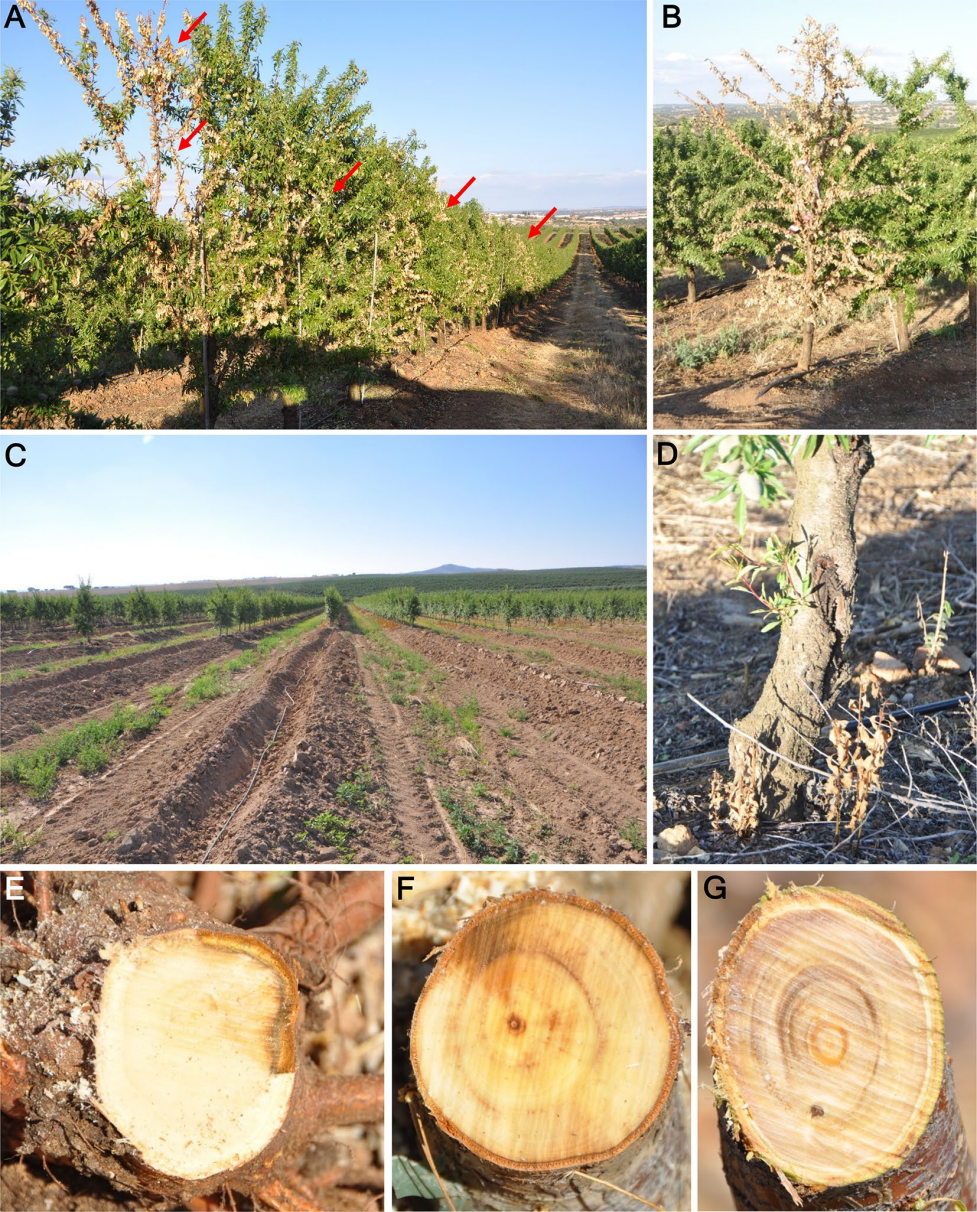
Symptoms of the disease in the field. (**A**) Row of almond trees showing wilting and leaf necrosis (red arrows); (**B**) dead tree; (**C**) affected area in the orchard that was uprooted after tree death; (**D**) canker lesions in the base of the trunk in an affected tree; **E**, cortical necrosis in the roots, ascending to the trunk; (**F**,** G**) vascular discoloration and necrosis in the trunk and branches caused by the pathogen infection.

All the trees of the Portuguese orchard were affected with different degrees of disease severity, ranging from partial wilting to death of trees. The trees showed wilting and leaf necrosis, gum secretion and canker formation in the trunk, and occassionally death (Fig. 1A–D). Internal symptoms were observed in an ascending direction from the root towards the trunk (Fig. 1E), showing necrosis and xylem discoloration (Fig. 1F,G). Only *Fusarium*-like colonies were consistenly isolated from the affected tissues from branches, trunk and roots with a frequency of isolation of 80.5, 84.0 and 64.6%, respectively.

Regarding the *Fusarium* isolates from southern Spain, they were collected from almond trees showing ADS in orchards throughout Andalusia region (southern Spain), and were isolated together with a wide diversity of fungal trunk pathogens causing co-infections in the same plant. The consistence of isolation was 32.0, 20.0, 78.5, 19.0 and 20.0% for *F. nirenbergiae* PV-1046, *F. proliferatum* PV-814, *F. redolens* PV-600, *F. sambucinum* PV-572, and *Fusarium* sp. A PV-747, respectively. In the orchards where healthy or asymptomatic plants were found, they did not show any symptoms of xylem discoloration and necrosis in the trunk.

### Molecular identification

**Figure 2:**
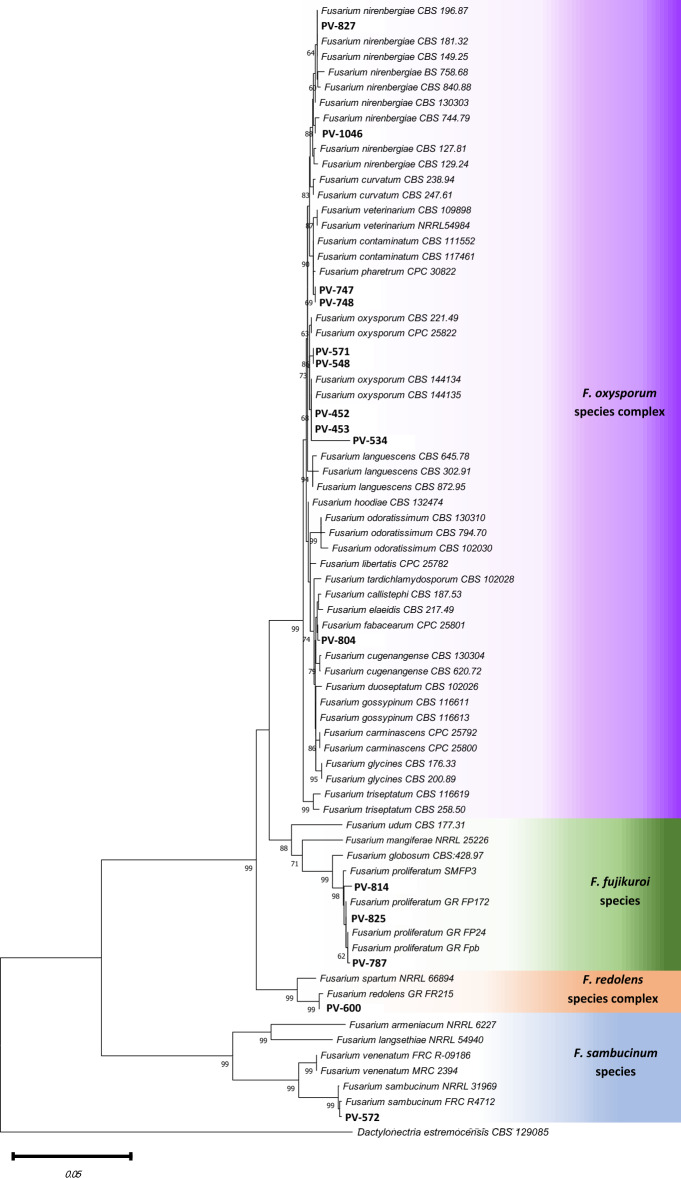
Phylogenetic inference of representative *Fusarium* isolates obtained from almond orchards in southern Spain and Portugal compared to reference strains of closely related species. The tree was inferred from a combined data set of *tef*1 and *rpb*2 sequences and rooted with *Dactylonectria estremocensis* (CBS 129085). Numbers below branches represent maximum parsimony bootstrap values from 2000 replicates. Isolates used in this study are highlighted in bold and species complexes are delimited by color.

The phylogenetic analysis using both *translation elongation factor *1*-α* (*tef*1) and *RNA polymerase II subunit* (*rpb*2) barcodes confirmed the identity of the 15 *Fusarium* isolates included in the data set (Fig. 2), compared to reference sequences of *Fusarium* species from GenBank (www.ncbi.nlm.gov). The three isolates from Portugal (PV-452, PV-453 and PV-534) and two isolates from Spain (PV-548, PV-571) clustered together with reference sequences of *F. oxysporum sensu stricto (s.s.)*. Within *F. oxysporum* species complex, the isolates PV-827 and PV-1046 were identified as *F. nirenbergiae*; whereas the isolates PV-747 and PV-748 or PV-804 were considered unidentified species and they were named as *Fusarium* sp. A or *Fusarium* sp. B, respectively (Supplementary Table S1). The isolates PV-787, PV-814 and PV-825 were identified as *Fusarium proliferatum* (*F. fujikuroi* species complex), the isolate PV-600 as *F. redolens s.s.* (*F. redolens* species complex), and the isolate PV-572 as *F. sambucinum* *s.s.* (*F. sambucinum* species complex).

### Phenotypic characterization

**Figure 3:**
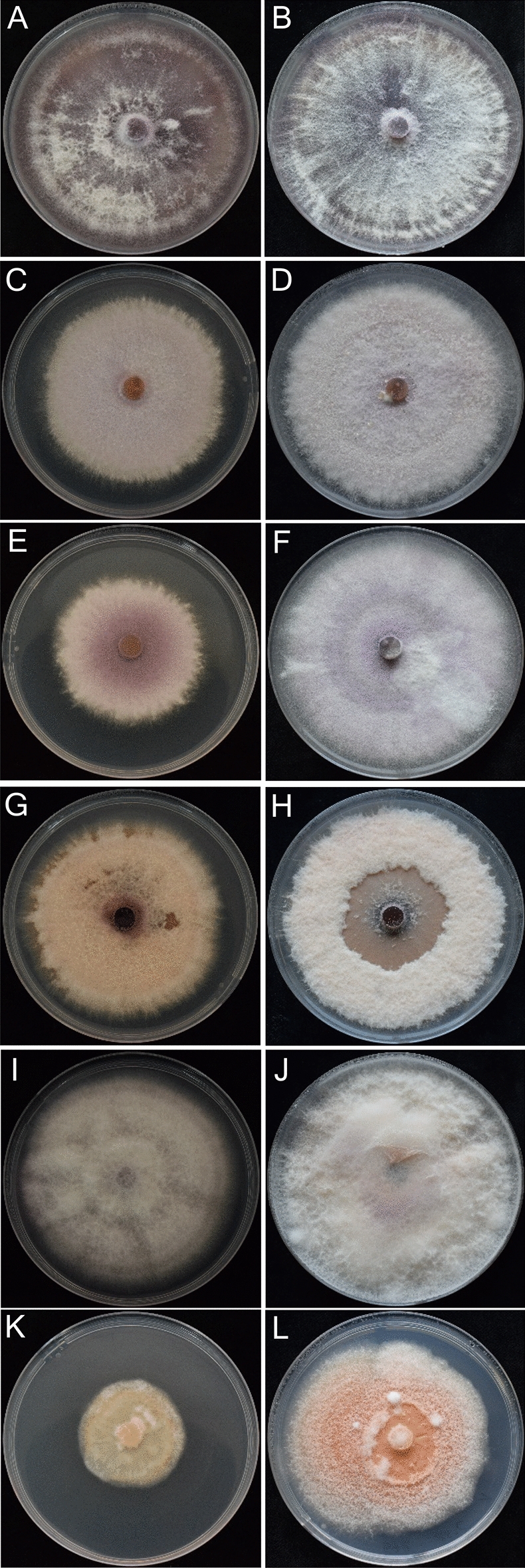
One- (left column) and two- (right column) week-old colonies grown on PDA at 25 ± 2 °C in the dark of the following *Fusarium* isolates: (**A**,** B**) *Fusarium* sp. A PV-747; (**C**,** D**) *Fusarium nirenbergiae* PV-1046; (**E**,** F**) *F. oxysporum* PV-534; (**G**,** H**) *F. proliferatum* PV-814; (**I**,** J**) *F. redolens* PV-600; and (**K**,** L**) *F. sambucinum* PV-572.

**Figure 4:**
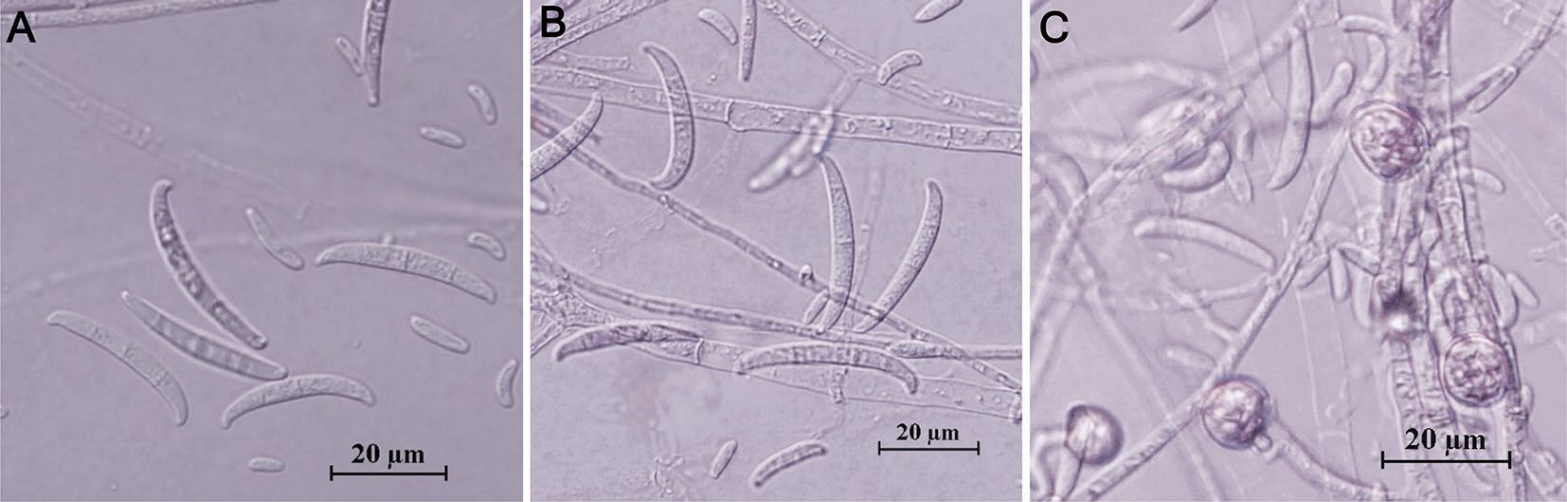
Micro- and macroconidia (**A**,** B**) and chlamydospores (**C**) of *Fusarium oxysporum* PV-534 developed on SNA at 25 ± 2 °C under continuous fluorescent light for 14 days.

For all isolates tested, colony morphology was described on potato dextrose agar (PDA; Fig. 3) and micro- and macroconidia characteristics on Synthetischer Nährstoffarmer Agar (SNA). Micro- and macroconidia dimensions are shown in Table 1. Colony morphology of the *Fusarium* isolates belonging to *F. oxysporum* species complex (PV-534, PV-747, PV-1046) varied widely. Mycelia was floccose, sparse or abundant, irregular in the margin, without zonation, with color ranging from white to pale violet. Violet or magenta pigmentation in the agar was not observed for any of the tested isolates (Fig. 3A–F). Microconidia were abundant, oval, elliptical or reniform, and usually 0-septate. Macroconidia were falcate to almost straight, thin walled, usually 3-septate, with length and width averages (*n* = 30) being 32.6 × 3.7, 16.6 × 4.0 and 14.6 × 3.6 μm for PV-534, PV-747 and PV-1046, respectively (Fig. 4A,B). Colony morphology of *F. proliferatum* PV-814 showed abundant aerial mycelium initially white but become slight purple-violet with age. Colonies showed regular margin and no zonation (Fig. 3G,H). Microconidia were usually formed in chains, 0-septate, and club shaped with a flattened base. Macroconidia were slender, almost straight, usually 3- to 5- septate, with length and width averages (*n* = 30) being 48.2 × 3.1 μm. The isolate *F. redolens* PV-600 showed colonies with relatively flat aerial mycelium that was white to pink in color, regular margin and without zonation (Fig. 3I,J). Microconidia were cylindrical, 0- to 1-septate and often pointed on one end. Macroconidia were robust, thick-walled widest towards the apical end, 3- to 5- septate with 3-septate the most frequent, with a hooked apical cell and a food-shaped basal cell. The length and width averages (*n* = 30) were 12.3 × 3.9 μm. Colonies of *F. sambucinum* PV-572 showed abundant aerial mycelia, floccose to felt-like and white to yellow to salmon-orange in color. The colonies showed a series of apparent concentric rings with lobed margins, and without zonation (Fig. 3K,L). Microconidia were oval and 0- to 1- septate, but they were not abundant. Macroconidia were slender, 3- to 5-septate, and falcate with a pointed apical cell and a food-shaped basal cell, with length and width averages being 36.9 × 5.3 μm. Chlamydospores were not observed in any case, except for *F. oxysporum* PV-534 (Fig. 4C).

**Table 1:** Measurements of micro- and macroconidia of the representative *Fusarium* isolates used in this study.

*Fusarium* species	Isolate	Microconidia		Macroconidia	
		Length × width (µm)	Length/width	Length × width (µm)	Length/width
*F. nirenbergiae*	PV-1046	(4.4-) 6.1 (-7.7) × (1.9-) 2.4 (-3.2)	2.5 ± 0.05	(9.6-) 14.6 (-20.5) × (2.8-) 3.6 (-5.1)	4.1 ± 0.12
*F. oxysporum*	PV-452	(4.7-) 8.8 (-14.9) × (1.9-) 2.7 (-3.8)	3.3 ± 0.15	(19.7-) 28.2 (-39.7) × (2.4-) 3.6 (-4.7)	7.8 ± 0.25
	PV-453	(4.5-) 8.6 (-14.7) × (1.7-) 2.6 (-3.7)	3.4 ± 0.13	(17.8-) 26.1 (-36.7) × (2.4-) 3.5 (-4.6)	7.4 ± 0.23
	PV-534	(5.5-) 9.5 (-14.7) × (2.0-) 2.9 (-3.9)	3.3 ± 0.11	(21.3-) 32.6 (-43.5) × (2.5-) 3.7 (-4.8)	8.7 ± 0.34
*F. proliferatum*	PV-814	(6.2-) 7.3 (-9.0) × (2.3-) 2.8 (-3.1)	2.6 ± 0.04	(34.8-) 48.2 (-60.6) × (2.2-) 3.1 (-4.0)	15.8 ± 0.48
*F. redolens *sensu stricto	PV-600	(6.4-) 7.7 (-9.2) × (2.5-) 3.2 (-3.8)	2.4 ± 0.05	(8.6-) 12.3 (-16.3) × (3.0-) 3.9 (-5.2)	3.2 ± 0.11
*F. sambucinum *sensu stricto	PV-572	(3.2-) 5.1 (-6.8) × (10.0-) 2.7 (-3.6)	1.9 ± 0.03	(24.9-) 36.9 (-57.0) × (3.7-) 5.3 (-7.5)	7.1 ± 0.17
*Fusarium* sp*.* A	PV-747	(5.4-) 7.6 (-10.0) × (2.7-) 3.1 (-3.7)	2.5 ± 0.05	(13.3-) 16.6 (-21.1) × (2.4-) 4.0 (-5.7)	4.2 ± 0.14

Mean and range values (in brackets) are indicated for Length × Width (µm). For both micro- and macroconidia, the values of each parameter represent the average of 30 conidia.

### Effect of temperature on mycelial growth

There were significant differences in both optimal growth temperature and mycelial growth rate (MGR; *P* ≤ 0.0001 in all cases) parameters among *Fusarium* spp. isolates. Most of them were able to slightly growth at both extreme temperatures (5 °C or 35 °C) except the isolates *F. nirenbergiae* PV-1046 and *Fusarium* sp. A PV-747 that showed 9.0 and 8.0 °C as minimum growth temperature, respectively; and for the isolates *F. nirenbergiae* PV-1046, *F. redolens* PV-600 and *F. proliferatum* PV-814 that showed 30.5, 31.0 and 32.0 °C as maximum growth temperature, respectively, according to the adjusted Analytics Beta model. The optimal growth temperature ranged from 26.4 to 16.8 °C for the *F. oxysporum* PV-453 and *F. sambucinum* PV-572, respectively. *Fusarium* isolates showed a MGR ranging from 5.4 to 3.1 mm day^-1^ for the *Fusarium* sp. A isolate PV-747 and *F. sambucinum* PV-572, respectively (Table 2).

**Table 2:** Effect of temperature on mycelial growth of the representative *Fusarium* isolates selected in this study grown on PDA at 5, 10, 15, 20, 25, 30 and 35 °C in the dark for 7 days.

*Fusarium* species	Isolate	Analytics Beta model			Temperature (°C)			MGR (mm day^−1^)
		*R*^2^	a	b	Optimum	Minimum	Maximum	
*F. nirenbergiae*	PV-1046	0.9987	1.18	0.44	24.7 ± 0.08 d	9.0	30.5	4.5 ± 0.09 d
*F. oxysporum*	PV-452	0.9992	2.36	1.06	25.7 ± 0.10 b	5.0	35.0	5.1 ± 0.04 ab
	PV-453	0.9983	1.73	0.70	26.4 ± 0.27 a	5.0	35.0	4.6 ± 0.14 d
	PV-534	0.9974	2.29	0.95	26.1 ± 0.10 ab	5.0	35.0	4.7 ± 0.23 cd
*F. proliferatum*	PV-814	0.9999	2.58	0.92	24.8 ± 0.02 cd	4.5	32.0	4.5 ± 0.09 d
*F. redolens sensu stricto*	PV-600	0.9830	2.02	0.58	25.0 ± 0.13 c	4.5	31.0	5.0 ± 0.09 bc
*F. sambucinum sensu stricto*	PV-572	0.9114	2.35	3.70	16.8 ± 0.11 f	4.0	37.0	3.1 ± 0.03 e
*Fusarium* sp. A	PV-747	0.9969	1.82	1.38	23.4 ± 0.11 e	8.0	35.0	5.4 ± 0.13 a

Data represent the average of eight replicated Petri dishes per isolate and temperature combination, obtained using Analytics Beta model, where *R*^2^ = coefficient of determination, and a, b = regression coefficients. For each isolate, temperature-averaged growth rates were fitted to a regression curve to estimate the optimal growth temperature, and the maximum growth rate (MGR; mm day^−1^) was obtained by the Analytics Beta model at the optimal growth temperature. Means in a column followed by a common letter do not differ significantly according to Fischer’s protected LSD test at *P* = 0.05^41^.

### Pathogenicity tests

#### *Fusarium oxysporum* PV-534 infections

**Figure 5:**
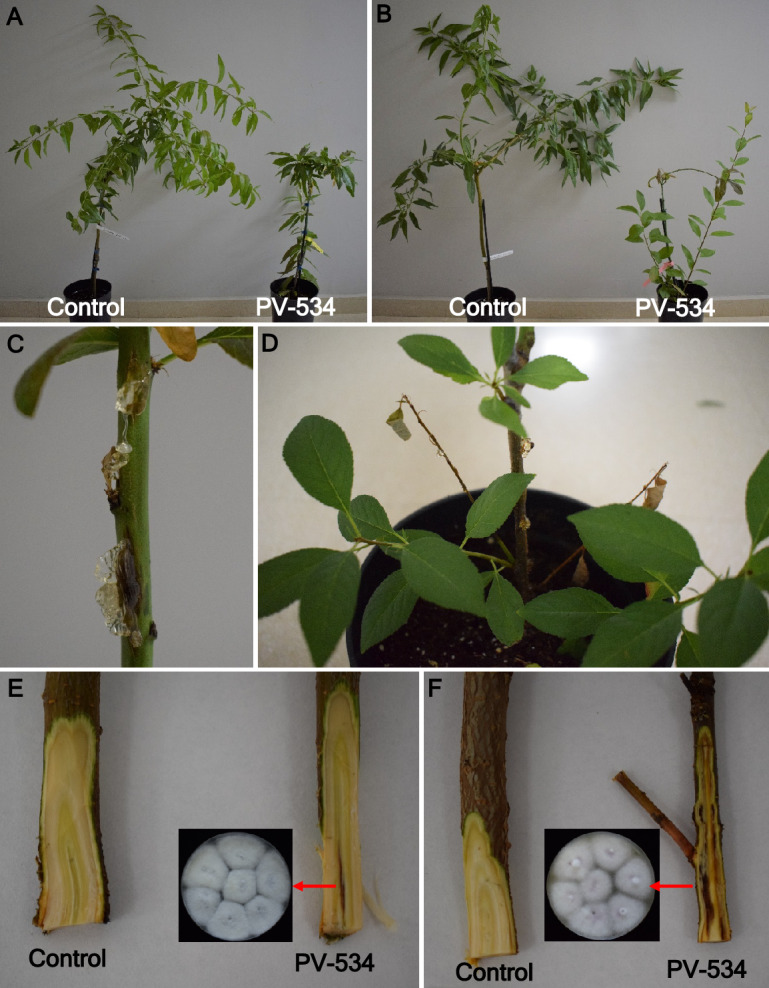
Symptoms developed in almond plants inoculated with the *Fusarium oxysporum* PV-534 isolate 6 months after inoculation. (**A**,** B**) Effect of the pathogen on the growth of almond plants of the cv. Lauranne grafted on GF-677 (**A**) or R20 (**B**) rootstocks; (**C**,** D**) gummosis, cankers and wilting in almond plants of the cv. Lauranne grafted on R20 rootstock; (**E**,** F**) necrosis and discoloration of the xylem in almond plants of the cv. Lauranne grafted on GF-677 (**E**) or R20 (**F**) rootstocks, and consistent reisolation of the pathogen in PDA.

Symptoms of the disease at six months after inoculation consisted in major plant growth reduction regardless the rootstock. However, almonds grafted on R20 rootstock developed gummosis and cankers through the stem showing higher internal necrotic lesions and discolored areas than in almonds grafted on GF-677 (Fig. 5). Inoculated plants showed a significant less growth (*P* ≤ 0.05) than noninoculated plants, with almonds grafted on R20 rootstock being significantly more susceptible to the infection than those grafted on GF-677. For the total length (cm) and fresh weigh of shoots, there were significant differences between treatments (*P* ≤ 0.0001; *P* = 0.0006, respectively) and for the interaction treatment × rootstock (*P* = 0.0068; *P* = 0.0023, respectively). Regarding total length of shoots, no significant differences were observed between rootstocks in non-inoculated control plants (Total Length_GF-677_ = 226.14 ± 33.4 cm; Total Length_R20_ = 261.6 ± 33.7 cm), whereas inoculated plants showed a shorter shoot length than noninoculated plants, this parameter being significantly lower on R20 (24.2 ± 2.4 cm) than in GF-677 (98.9 ± 16.9 cm). For shoot fresh weight, only the inoculated plants grafted on R20 showed a significantly lower shoot fresh weight (17.6 ± 4.8 g) compared to non-inoculated plants (Fresh weight_GF-677_ = 91.1 ± 12 g; Fresh weight_R20_ = 106.9 ± 9.1 g) or to inoculated plants grafted on GF-677 (75.8 ± 10.3 g). Regarding the sprouting of buds from the stems, and the total length and fresh weight of the new shoots, noninoculated plants grafted on GF-677 did not produce any shoots. However, the pathogen infection significantly induced bud sprouting, with inoculated plants grafted on R20 showing significantly higher bud sprouting, and lesion length and fresh weight of new shoots compared to the inoculated plants grafted on GF-677 and to the non-inoculated plants (Table 3). The pathogen was reisolated from the stem and from the wood and roots of the rootstock in the inoculated plants grafted on R20. It was also isolated from the wood and roots of the rootstock in the inoculated plants grafted on GF-677, but the pathogen was not reisolated from any tissues of the noninoculated plants, regardless of the rootstock (Table 4).

**Table 3:** Effect of *Fusarium oxysporum* PV-534 infections on growth of almond plants of the cv. Lauranne grafted on GF-677 or R20 rootstocks six months after inoculation.

Treatment	Rootstock	Shoots		New shoots from sprouted buds		
		Length (cm)	Fresh weight (g)	Number	Length (cm)	Fresh weight (g)
Control	GF-677	226.14 ± 33.4 a	91.1 ± 12.3 a	0.0 ± 0.0 c	0.0 ± 0.0 c	0.0 ± 0.0 c
	R20	261.6 ± 33.7 a	106.9 ± 9.1 a	0.8 ± 0.2b	18.6 ± 6.1 b	12.4 ± 8.5 b
*F. oxysporum* PV-534	GF-677	98.9 ± 16.9 b	75.8 ± 10.3 a	0.6 ± 0.4b	10.9 ± 8.1 b	5.8 ± 4.3 b
	R20	24.2 ± 2.4 c	17.6 ± 4.8 b	3.4 ± 0.5 a	79.5 ± 12.4 a	28.6 ± 3.5 a

Data represent the average of 12 replicated plants per treatment combination. Means in a column followed by the same letter do not differ significantly according to Fisher’s protected LSD test at *P* = 0.05^41^.

**Table 4:** Frequency of reisolation (%) of fungal isolates used in each pathogenicity test.

Pathogenicity tests	Frequency of reisolation (%)					
	GF677			R20		
	Stem	Wood	Root	Stem	Wood	Root
*Fusarium oxysporum* PV-534 infections						
Control plants	0.0	0.0	0.0	0.0	0.0	0.0
Inoculated plants	0.0	23.8	14.3	42.9	37.5	16.1
Effect of environmental conditions on *Fusarium oxysporum* PV-534 infections						
Control-Irrigation	0.0	–	0.0	0.0	–	0.0
Control-Dry	0.0	–	0.0	0.0	–	0.0
Inoculated plants-Irrigation	66.7	–	90.5	83.3	–	69.0
Inoculated plants-Dry	50.0	–	64.3	47.6	–	59.6
Infections using natural infested soil						
Control (sterilized soil)	0.0	0.0	0.0	0.0	0.0	0.0
Natural infested soil	0.0	14.3	36.5	0.0	23,8	58.7
Pathogenicity of *Fusarium* spp. strains from southern Portugal and Spain						
Control	0.0	–	0.0	–	–	–
Inoculated plants						
* F. nirenbergiae* PV-1046	97.6	–	95.3	–	–	–
* F. oxysporum* PV-534	66.7	–	90.5	–	–	–
* F. proliferatum* PV-814	71.4	–	100	–	–	–
* F. redolens sensu stricto* PV-600	50.0	–	92.9	–	–	–
*F. sambucinum sensu stricto* PV-572	59.5	–	78.6	–	–	–
*Fusarium* sp. A PV-747	71.5	–	87.9	–	–	–

In each pathogenicity test (experiment), reisolations of three plants were carried out for each combination of treatments and rootstocks. For each treatment and plant tissue combination, three Komada Petri dishes were seeded with seven pieces of tissue per plate. Reisolation percentage was calculated as [(nº of positive seeded points/nº of total attempts of isolation) × 100].

#### Effect of environmental conditions on * Fusarium oxysporum* PV-534 infections

**Figure 6:**
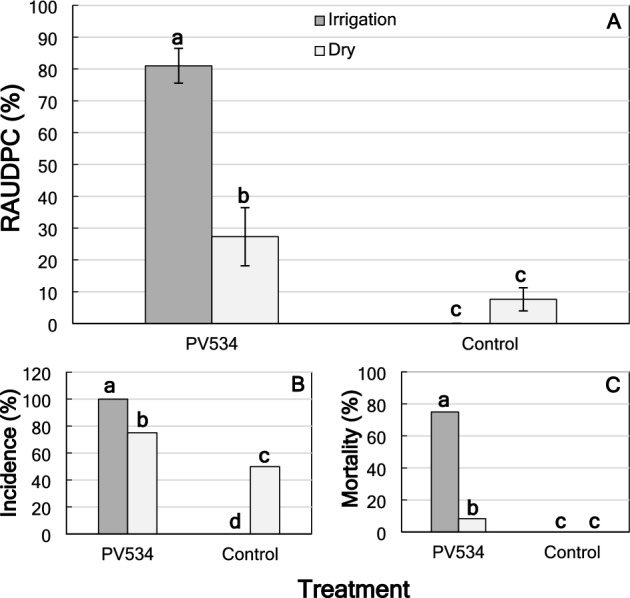
(**A**) Disease severity (Relative area under the disease progress curve; RAUDPC, %) for the interaction treatment (inoculated or noninoculated control plants) × environment (irrigation or dry) in almond plants of the cv. Lauranne 3 months after inoculation with *Fusarium oxysporum* PV-534. Columns represent the means of 24 replicated plants. The vertical bars are the standard error of the means. Columns with the same letter do not differ significantly according to Fisher’s protected LSD test at *P* = 0.05^41^; (**B**,** C**) Incidence (%) and mortality (%) for the interaction treatment × environment at the end of the experiment. Columns represent the means of 24 replicated plants. Columns with by the same letter do not differ significantly according to multiple comparisons for proportions tests at *P* = 0.05^45^.

Wilt symptoms were observed in the inoculated plants under the two environmental conditions studied starting 2 weeks after inoculation, regardless of the rootstock used. There were significant differences between treatments (control or inoculated plants), environmental conditions and for their interaction (*P* ≤ 0.0001). However, there were no significant differences between rootstocks (*P* = 0.7887), and the interactions treatment × rootstock (*P* = 0.7842), rootstock × environment (*P* = 0.9796) and treatment × rootstock × environment (*P* = 0.5711). Thus, only the treated means of the interaction treatment × environment were analyzed. Disease severity (DS) was significantly higher in inoculated plants under irrigation [Relative area under the disease progress curve (RAUDPC) = 81.0 ± 5.5%] than inoculated plants under dry conditions (RAUDPC = 27.3 ± 9.1%) (Fig. 6). Control plants did not show symptoms related with the disease regardless of the environmental conditions. The inoculated plants under irrigation showed significantly highest incidence (100%) and mortality (75.0%) compared to those under dry conditions (incidence = 75%; mortality = 8.3%). The pathogen was reisolated from the stem and roots of inoculated plants, but not from any tissue of non-inoculated plants, regardless of rootstock or environmental conditions (Table 4).

#### Infections using natural infested soil

One year after inoculation, plants grown in both sterile soil and naturally infested soil showed no external symptoms. However, almonds grafted on R20 growing in natural infested soils showed xylem necrosis in an ascending direction from the roots to the stem. Nevertheless, no internal symptoms were observed in almonds grafted on GF-677 growing in naturally infested soils, or in plants growing in sterilized soil regardless of the rootstock. Roots did not show any symptoms regardless of the rootstock and the type of soil. The pathogen was reisolated from the wood and roots of the rootstock in the inoculated plants grafted on both GF-677 and R20 rootstock, but the pathogen was not reisolated from the stem or any tissue of the noninoculated plants, regardless of the rootstock (Table 4). The final propagule density in the naturally infested soils was 97.2 colony forming units (CFUs) per g of soil.

#### Pathogenicity of *Fusarium* spp. strains from southern Portugal and Spain

**Figure 7:**
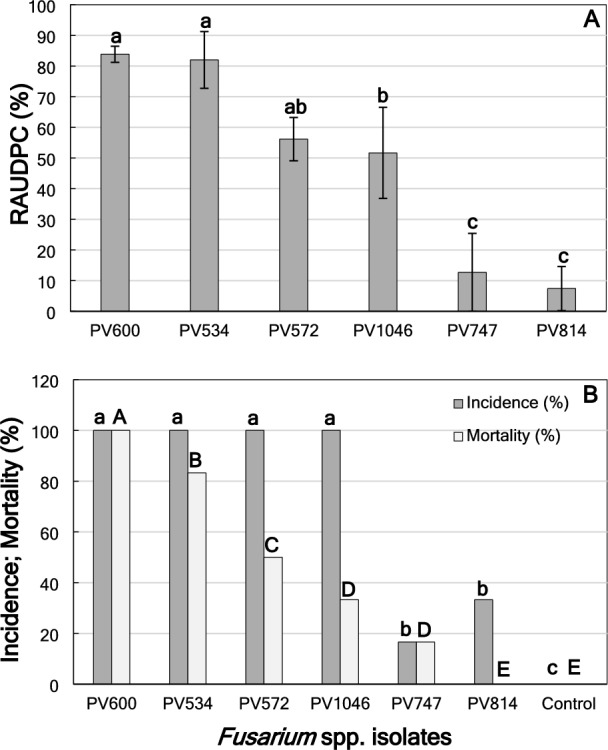
(**A**) Disease severity (Relative area under the disease progress curve; RAUDPC, %) in almond plants of the cv. Lauranne grafted on GF677 at 3 months after inoculation with the isolates *F. nirenbergiae* PV-1046, *F. oxysporum* PV-534, *F. proliferatum* PV-814, *F. redolens* PV-600, *F. sambucinum* PV-572, and *Fusarium* sp. A PV-747. Columns represent the means of 12 replicated plants. The vertical bars are the standard error of the means. Columns with the same letter do not differ significantly according to Fisher’s protected LSD test at *P* = 0.05^41^; (**B**) Incidence (dark gray columns; %) and mortality (light gray columns; %) at the end of the experiment. Columns represent the means of 12 replicated plants. Columns with the same lower or capital letter do not differ significantly according to multiple comparisons for proportions tests at *P* = 0.05^45^ for incidence and mortality, respectively.

There were significant differences (*P* ≤ 0.0001) in DS between *Fusarium* species in almond plants at 3 months after inoculation. *Fusarium redolens* PV-600 (RAUDPC = 83.9 ± 2.6%) and *F. oxysporum* PV-534 (RAUDPC = 82.0 ± 9.2%) were the most aggressive species followed by *F. sambucinum* PV-572 (RAUDPC = 56.2 ± 7.0%) and *F. nirenbergiae* PV-1046 (RAUDPC = 51.7 ± 14.9%). *Fusarium* sp. A PV-747 (RAUDPC = 12.7 ± 12.7%) and *F. proliferatum* PV-814 (RAUDPC = 7.4 ± 7.2%) were the least aggressive species (Fig. 7A). Plants inoculated with *F. redolens* PV-600, *F. oxysporum* PV-534, *F. sambucinum* PV-572 and *F. nirenbergiae* PV-1046 showed 100% of incidence, but only *F. redolens* PV-600 showed 100% of mortality. *Fusarium proliferatum* PV-814 (incidence = 33.3%) and *Fusarium* sp. A PV-747 (incidence = 16.6%) showed the least incidence, and no mortality was observed for *F. proliferatum* PV-814 (Fig. 7B). The pathogen was reisolated from the stem and roots from plants inoculated with all six *Fusarium* species, but the pathogen was not reisolated from any tissue of noninoculated plants, regardless of the rootstock (Table 4).

## Discussion

In this study, we demonstrate that several *Fusarium* species recovered from symptomatic trees are pathogenic to almond, causing crown rot and necrotic lesions to the xylem vessels as well as associated with the complex ADS. Canker diseases in almond associated with *Fusarium* spp. are poorly understood, even though their role associated with trunk diseases is a current point of debate in several perennial crops such as avocado, citrus, grapevine or tree nuts^2,7,9,11,12,14^. To date the species *Fusarium acuminatum, F. avenaceum, F. brachygibbosum, F. californicum, F. euwallaceae*, and *F. solani* has been recovered from almonds showing cankers and vascular streaking worldwide^5,19,22,23^. In addition, *F. oxysporum* has isolated from almond hulls in California producing large quantities of styrene and isomers of 7-methyl-1,3,5-cyclooctatriene^24^. Therefore, this present work was conceived to shed light on the identification and pathogenic role of *Fusarium* species causing cankers and almond decline to improve the knowledge on this matter of study.

The three Portuguese isolates were identified as *F. oxysporum*, and the identity of the Spanish isolates was confirmed as *F. nirenbergiae*, *F. oxysporum, F. proliferatum*, *F. redolens*, *F. sambucinum*, and *Fusarium* sp. in concordance with the previous molecular identification done by Antón-Domínguez et al.^1^. These authors recovered all these *Fusarium* species from almond trees showing ADS in Spain, even though with low consistency of isolation as well as in association with a broad diversity of fungal trunk pathogens including species that belongs to *Botryosphaeriaceae*, *Dyaporthaceae* and *Diatrypaceaea* mainly^1^. However, only the pathogenicity of *F. oxysporum* isolate PV-548 from Spain was tested in detached almond shoots showing minimal aggressiveness, whereas the pathogenic role of the remaining *Fusarium* isolates including those from Portugal was still absent.

Colony and conidia characteristics were useful to confirm their identity since they were in concordance with their corresponding taxonomic description^6,25^. Regarding the effect of temperature on mycelial growth, the three *F. oxysporum* isolates from Portugal were able to growth from 5 to 35 °C, and showed an optimal growth temperature around 26 °C and a MGR around 5 mm day^−1^. The optimal growth temperature of the Portuguese isolates was significantly higher compared to the Spanish isolates that showed values ranging from 16.8 to 25.0 °C for *F. sambucinum* PV-572 and *F. redolens* PV-600, respectively. The behaviour on MGR was similar for almost all the isolates regardless of geographic origin, with the exception to *F. sambucinum* PV-572, which showed the lowest MGR (3.1 mm day^−1^) in the entire experiment. The low optimal growth temperature of *F. sambucinum* could be explained by its own genetics since this species is more common in temperate parts of the world, but can be also recovered from areas as far north as Iceland and northern Norway close the Arctic Circle^6^.

Due to *Fusarium* spp. are well known soilborne fungi, we used root dipping and soil inoculation methods to test the pathogenicity of our isolates according to the protocols required for these fungi^12,26–28^. The pathogenicity tests were focused on determining the pathogenic role of *F. oxysporum* PV-534 from Portugal because it was isolated in a high consistency from almond trees showing severe symptoms of decline and xylem discoloration with 100% of incidence. In addition, it was isolated alone unlike the Spanish isolates that were recovered associated with a great diversity of fungal trunk pathogens^1^. The global conclusions from pathogenicity tests provide not only evidence of the pathogenic role of *F. oxysporum* PV-534, but also that the rootstock R20 is more susceptible to the pathogen than GF-677. Notice no significant differences were observed in the growth of noninoculated almond plants regardless of the rootstock (GF-677 and R20), with no dwarfing effect of R20 being appreciated. Although the dwarfing effect of R20 is one of the main agronomic characteristics of this rootstock, it was not appreciated in the experiments of the present study probably due to root growth confined in pots. All the results obtained here were confirmed using both artificial inoculum and naturally infested soil in potted plants. Nevertheless, no differences in rootstock susceptibility were observed in the experiment evaluating the effect of environmental conditions on *F. oxysporum* infections, where plants under irrigation were significantly more susceptible than those under dry conditions. The different inoculum source in the experiments (conidial suspensions from PDA or conidial suspensions from PDB) could influence in the disease progression since symptoms in plants inoculated with suspension of PDA conidia appear later than in those inoculated with PDB conidia. In addition, difference in rootstock susceptibility was observed in plants inoculated with PDA conidia, but not in plants inoculated with PDB conidia. Furthermore, frequent irrigation could favor the establishment of the inoculum in the substrate and the development of infections, causing a higher DS than in poorly watered plants.

In the experiment testing all *Fusarium* strains from Portugal and Spain, *Fusarium oxysporum* PV-534 (*F. oxysporum* complex) and *F. redolens* PV-600 (*F. redolens* complex) were the most aggressive species to almond compared to the remaining *Fusarium* species and isolates tested; while *F. sambucinum* PV-572 (*F. sambucinum* complex) and *F. nirenbergiae* PV-1046 (*F. oxysporum* complex) showed a moderate aggressiveness, and *Fusarium* sp. A PV-747 (*F. oxysporum* complex) and *F. proliferatum* PV-814 (*F. fujikuroi* complex) were the least aggressive. All they can be considered pathogenic to almond, although with different levels of aggressiveness. Thus, our results suggest a high level of intra- and inter-specific variability in aggressiveness among species within *F. oxysporum* species complex, as well as among complexes of *Fusarium* species, which should be studied using biomolecular and bioinformatics tools. The wide diversity of *Fusarium* spp. showing different degrees aggressiveness has been already demonstrated in nuts, such as almond, pistachio and walnut^2,5,15–17,19^. Considering the two most aggressive species of this study, *F. oxysporum* is the most widely documented *Fusarium* species, and one of the most destructive species on herbaceous and perennial crops as well as in forest and ornamental plants causing vascular wilt, damping-off, and crown and root rots^6,14,20,29^; *F. redolens* has been associated with root rot diseases in a broad diversity of herbaceous host such as asparagus, beans, carnation, peas and spinach^6^, and more recently in woody crops such as *Panax ginseng*^30^ and *Panax quinquefolius*^31^.

Many *Fusarium* isolates appear to be host specific, and according to this character they are subdivided into *forma speciales* and race reflecting the apparent plant pathogenic specialization^6,25^. In addition, the pathogenicity of a particular *Fusarium* isolate in a certain host could be determined by a single effector gene that allow one small genetic transfer event between isolates to confer novel pathogenicity^32^. Thus, in the case of *F. oxysporum* PV-534 that showed higher specificity in almond showing wilting symptoms, a comparative genomic analysis between species and isolates with different levels of aggressiveness in almond and other woody crops would be useful to better understanding its pathogenic role in almond. In addition, determination of the production of polyketide secondary metabolites, with special emphasize on toxins^25^, would be also necessary to elucidate the genetic and functional mechanism of the infection process of this pathogen. Considering the scenario in the Portuguese almond orchard, we hypothesize that the infections were caused due to the high levels of inoculum of *F. oxysporum* in the soil because the history of *Fusarium*-susceptible herbaceous hosts grown in this soil over decades. Thus, determining the race of this isolate besides their mechanism of infection may help us to improve the knowledge on its pathogenic role.

Regarding the remaining *Fusarium* species tested in this study, at this point of the research, we cannot conclude that they are the causal agents of almond decline since they were recovered in association with several trunk pathogens from almonds showing ADS. In this case, we may consider a disease complex associated with a wide diversity of fungi that most of them probably play an endophytic behavior, including *Fusarium* species, and they may influence in both biotic and abiotic stresses^1,33^. According to this hypothesis, a recent study conducted in Canada by Úrbez-Torres et al.^12^ identifying *Fusarium* species associated with grapevine decline, reveals that *Fusarium* may be a secondary pathogen on grapevines since *Fusarium* spp. caused similar necroses in rootstock roots and basal ends than those caused by *Dactylonectria macrodidyma*, *D. pauciseptata* and *Ilyonectria liriodendri* (black foot pathogens belonging to *Nectriaceae*), but both fungi were not able to reduce significantly root and shoot dry weights compared with noninoculated controls in most of the treatments tested. In addition, these same authors suggested that *Fusarium* could enhance DS in wounded cuttings as well as in the presence of other grapevine trunk disease pathogens such as black foot fungi because they observed significantly higher DS when grapevine cuttings were co-inoculated with *Fusarium* spp. and *D. macrodidyma*^12^. Previous studies also revealed the potential interactions between the invasive *Fusarium circinatum*, causal agent of pitch canker in *Pinus* spp., and other important pathogens associated with canker diseases such as *Diplodia sapinea*, *Caliciopsis pinea*, *Cenangium ferruginosum* and *Gremmeniella abietina* in *Pinus* spp.^34,35^. Therefore, further research evaluating the interaction between *Fusarium* species and other fungal trunk pathogens such as *Botryosphaeriaceae* and *Diaporthe* species associated with ADS is required to determine their pathogenic role in almond.

## Conclusions

This present study demonstrated the pathogenic role of species belonging to *F. oxysporum* species complex causing almond decline, and create debate in the endophytic role of other *Fusarium* species within *F. fujikuroi*, *F. oxysporum*, *F. redolens*, and *F. sambucinum* species complexes. On the one hand, *F. oxysporum* is the causal agent of the wilting dieback and canker syndrome observed in the Portuguese orchard since it was the only fungal species recovered from the affected tissues and its pathogenicity was demonstrated. On the other hand, the *Fusarium* species from Spanish almond orchards, which were isolated together with other canker pathogens, showed different levels of aggressiveness and their potential interaction with other almond canker pathogens should be determined in future studies.

## Materials and methods

### Field surveys and fungal isolation

In spring 2017, symptoms of wilting, dieback, and cankers with gum secrection were observed affecting severely to a 3-years-old almond orchard of cv. Lauranne grafted on R20 rootstock located in Monforte (southern Portugal). Twelve affected trees were uproated and sectioned. Samples of roots, trunk and branches were collected, kept into plastic bags and brought to the Department of Agronomy at the University of Cordoba (UCO; Spain). Each sampled wood section was processed individually for fungal isolation. To this end, the bark was removed, whased under runing tap water, and superficially disinfected by spraying the sample with a 70% (vol/vol) alcohol solution and flaming. Small wood pieces from the margin of necrotic areas in each plant tissue were cut with a sterile scalpel and placed onto PDA (Difco Laboratories Inc., Detroit, U.S.A.) acidified with lactic acid [APDA; 1 ml of 25% (vol/vol) per liter of medium; pH = 4.0–4.5] to minimize bacterial contamination. From each plant tissue per tree, 21 wood pieces were collected and placed on three Petri dishes filled with APDA, at a rate of 7 wood pieces per plate. Thus, a total of 756 wood pieces were used as attempts of isolation (inoculation points on Petri dishes: 12 trees × 3 plant tissues × 3 Petri dishes × 7 wood pieces per Petri dish). Petri dishes were incubated at 23 ± 2 °C in the dark for 21 days. They were periodically observed, and the growing colonies were transferred to PDA and incubated as described before. Only *Fusarium*-like colonies were isolated, and the average of the frequency of isolation (%) was estimated per each wood section. Notice that the isolation attempts (wood pieces onto APDA without fungi) where *Fusarium*-like colonies appeared, were removed from the original Petri dishes immediately after transferring to PDA. The wood pieces without fungi were kept in APDA to wait for the isolation of other potential trunk pathogens with a slower growth rate, such as species of *Diatrypaceae*, *Phaeomoniella*, *Cadophora* or *Phaeoacremonium*, among others. However, after 3 weeks of incubation, any other fungi were isolated.

### Fungal isolates

Based on colony morphology and pigmentation, three groups of *Fusarium*-like isolates collected from almond trees from Portugal were observed and one representative isolate per group (PV-452, PV-453, PV-534) was selected for further analysis in this study. In addition, 12 *Fusarium* isolates associated with ADS in southern Spain already identified by Antón-Domínguez et al.^1^ were included in this study to confirm their molecular identification, and specially to test their pathogenicity by comparing their aggressiveness with those from almond trees in Portugal (Supplementary Table S1). Molecular and morphological identification of the isolates was performed with monosporic isolates previously obtained from each mass isolate using the serial dilution method described by Dhingra and Sinclair^36^. They are stored in 15% glycerol solution at − 80 °C in cryovials in the fungal collection of the Department of Agronomy at the UCO. Prior to use, the isolates were first grown on APDA and incubated at 23 ± 2 °C in the dark for 7 days. Growing fungal colonies were transferred to PDA and incubated under the same conditions.

### Molecular identification

The three *Fusarium* isolates from Portugal (Supplementary Table S1) were grown on PDA as described before, and genomic DNA was extracted from fresh grinded mycelium using the protocol of the E.Z.N.A Fungal DNA commercial kit (OMEGA BioTek, Norcross, GA, USA). The *tef*1 and *rpb*2 regions were amplified with the primer pairs EF1-728F/EF1-986R^37^ and fRPB2-5F/fRPB2-7cR^38^, respectively. The PCRs were performed in a total volume of 25 µl containing 20 ng of genomic DNA, 5 µl of 5 × My Taq Reaction Buffer and 0.13 µl of My Taq DNA Polymerase (Bioline). Each primer was used at 0.4 μM. A negative control was included in all PCR runs, using ultrapure water instead of DNA. The PCR cycling programs were as follows: for *tef*1: initial denaturation at 95 °C for 3 min, followed by 30 cycles of 94 °C for 30 s, 48 °C for 30 s and 72 °C for 1 min, and a final extension at 72 °C for 10 min; for *rpb*2: initial denaturation at 95 °C for 3 min, followed by 35 cycles of 94 °C for 15 s, 55 °C for 20 s and 72 °C for 1 min, and a final extension at 72 °C for 7 min. PCR products were purified following the protocol of the MEGAquick-spin™ Total Fragment DNA Purification commercial kit (INTRON Biotechnology), and they were submitted for sequencing to the Central Service Support Research (SCAI) of the University of Cordoba (Spain). Resulting forward and reverse sequences were assembled using SeqMan software (DNASTAR Lasergen SeqMan v. 7.0.0, Madison, WI, USA.), and consensus sequences were compared with the NCBI nucleotide by means of BLAST (http://ncbi.nlm.nih.gov/Blast.cgi). Reference sequences with the highest matches (> 98%) were used to perform a sequence database (Supplementary Table S1). *tef*1 and *rpb*2 sequences of *Fusarium* isolates from Spain^1^ (Supplementary Table S1) were also included in the sequence database. The sequences of *Fusarium* isolates from Portugal were deposited in GenBank, and their GenBank accession numbers are shown in Supplementary Table S1. A neighbour-joining analysis using the *tef*1 and *rpb*2 sequences alone (*Data not shown*) was conducted by means of the maximum composite likelihood method with 2000 bootstrap replicates, and genetic distances were estimated using the Kimura 2-parameter mode using MEGA11^39^. Alignments were concatenated manually by consolidating both alignments, and the combined alignment was used to infer phylogeny by means of maximum parsimony methods and bootstrapped 2000 times using MEGA11^39^.

### Phenotypic characterization

Based on the molecular identification, representative isolates of the different *Fusarium* species complex identified were selected (*n* = 8) to complete the phenotypic characterization (Supplementary Table S1). All the isolates were first grown on PDA at 23 ± 2 °C in the dark for 14 days.

Texture, density, color, margin and zonation of mycelial colonies were observed at 7 and 14 days of incubation at 25 ± 2 °C in the dark^40^ and colony color was determined according to the Rayner’s color scale^41^. To induce conidiophore and conidia production, all the isolates were grown on SNA^6^ and incubated at 25 ± 2 °C under continuous fluorescent light for 14 days. Then, mycelial plugs from SNA were placed on slides adding a drop of 0.01% acid fuchsine in lactoglycerol (1:2:1, lactic acid:glycerol:water), and covered with a coverslip. Fungal structures were observed and measured at × 400 magnification using a Nikon Eclipse 80i microscope (Nikon Corp., Tokyo, Japan). For each isolate, 30 micro- or macroconidia were measured and the average length and width, as well as the length/width ratio, were calculated.

### Effect of temperature on mycelial growth

The eight representative isolates selected for this study (Supplementary Table S1) were grown on PDA as described above. Mycelial agar plugs of 7 mm in diameter were obtained from the margin of active growing colonies, plated on the center of Petri dishes filled with PDA, and incubated at 5, 10, 15, 20, 25, 30 or 35 °C in the dark for 7 days. Four Petri dishes per isolate and temperature combination were used, and arranged in a completely randomized design. The experiment was repeated once.

For each isolate and temperature combination, the largest and smallest diameter of the colony was averaged, and converted to mycelial radial growth rate (mm day^−1^). To evaluate the variation of mycelial growth rate over temperature, data of each isolate was subjected to a nonlinear adjustment by means of the generalized Analytics Beta model^42^, and the optimal growth temperature and maximum growth rate (MGR; mm day^−1^) of each isolate were estimated as described by López-Moral et al.^43^. For both optimal growth temperature and MGR parameters, the data from the two repetitions were combined after checking that there were no significant differences between them (*P* ≤ 0.05), and tested for homogeneity of variances. One-way ANOVA was conducted with optimal growth temperature or MGR as dependent variable and fungal isolates as independent variable. Mean comparison was conducted according to Fisher's LSD test at *P* = 0.05^44^. Data from this study were analyzed using Statistix 10 software^45^.

### Pathogenicity tests

#### Plant material

In all experiments described below, healthy one-year-old almond plants of cv. Lauranne grafted on GF-677 or R20 rootstocks growing in peat moss in PVC pots (0.5 l) were used. Plants were obtained from a commercial nursery and preconditioned in a greenhouse at 23 ± 2 °C for one month before inoculations, and irrigated three times per week. Four trials were performed as described below, and the experiments were conducted during spring–summer of two consecutive years.

#### Fungal isolates, inoculum preparation and inoculation

The isolate *F. oxysporum* PV-534 from Portugal, and the Spanish isolates *F. nirenbergiae* PV-1046, *F. proliferatum* PV-814, *F. redolens* PV-600, *F. sambucinum* PV-572, and *Fusarium* sp. A PV-747 (Supplementary Table S1) were used for pathogenicity tests. For inoculum preparation, the *Fusarium* isolates were grown on PDA as described above and two types of inoculum were used: i) conidial suspensions from 7-day-old PDA colonies were obtained using sterile distilled water (SDW) and adjusted at 10^5^ conidia ml^−1^ by means of a hemocytometer, and it was used for plant inoculation; ii) conidial suspensions of the pathogen were obtained as described above, and 500 µl of the conidial suspension were added into a 2 L Erlenmeyer flask filled with 1 L of sterile potato dextrose broth (PDB; Difco Laboratories, Detroit, U.S.A.) and incubated at 25 °C for 7 days under continuous fluorescent light in an orbital shaker at 90 rpm (Grant bio PSU-20i, Grant Instruments, Cambridge, UK). Then, the PDB inoculum was diluted in SDW to obtain a final concentration of 10^6^ conidia ml^−1^ based on hemocytometer counts, that was used for plant inoculation.

For inoculation, regardless of the types of inoculum, plants were carefully uprooted, and the roots were immersed in the final conidial suspension for 30 min. Subsequently, the inoculated plants were transplanted in PVC pots previously disinfested with a commercial bleach solution, and filled with sterile peat moss. The peat moss sterilization was conducted prior to inoculation in two consecutive days at 120 °C for 50 min (1st day) and 120 °C for 20 min (2nd day). After transplanting, each plant was irrigated with 300 ml of the inoculum, and plants were maintained in the dark at 100% relative humidity (RH) overnight. Subsequently, plants were maintained in a controlled-growth chamber [23 ± 2 °C, with a 12:12-h (light:dark) photoperiod of white fluorescent light (10,000 lx) and 60% RH], and they were irrigated three times per week until the end of the experiments.

#### *Fusarium oxysporum* PV-534 infections

Almond plants of the cv. Lauranne grafted on GF-677 or R20 rootstocks were inoculated with a conidial suspension of *F. oxysporum* sp. PV-534 obtained from PDA as described before. For each rootstock, plants transplanted and treated with only water were included as control. There were six replicated plants per rootstock and fungal isolate or control, and they were arranged in a completely randomized design.

Plants were maintained in a controlled growth chamber as described above. Plant growth development was estimated at six months after inoculation by measuring the total length of shoots (cm) and fresh weight (g); and the number of new sprouted buds, and the total length (cm) and fresh weight (g) of the new shoots. Plants were uprooted to observe the roots and the internal discoloration in the rootstock. Three inoculated or control plants were randomly selected for reisolation. Wood sections from the stem (cultivar), and wood sections and roots from the rootstock were collected for fungal reisolation that was conducted in Komada medium (Leslie and Summerell 2006). The frequency of isolation (%) was estimated as [(nº of positive inoculated points/nº of total attempts of isolation) × 100].

#### Effect of environmental conditions on * Fusarium oxysporum* PV-534 infections

Two lots of plants of the cv. Lauranne grafted on GF-677 or R20 rootstock were inoculated with conidial suspensions of *F. oxysporum* PV-534 obtained from PDB as described before. After inoculation, one lot of plants was maintained in greenhouse conditions at 23 ± 2 °C and irrigated three times per week; whereas the second lot of plants was maintained in a shadehouse during late spring–summer at environmental temperature (25–35 °C) and irrigated once a week to simulate stressed conditions. Lots of plants of each rootstock treated with only water were included as control under each environmental condition. For each environment, there were six replicated plants per rootstock and treatment combination (PV-534 or control), and they were arranged in a completely randomized design.

DS was periodically evaluated along 12 weeks after inoculation using a 0 to 16 rating scale adapted from López-Moral et al.^46^, and the RAUDPC was estimated at the end of the experiment by the trapezoidal integration method^47^. Incidence and mortality were also assessed at the end of the experiment as the percentage of symptomatic or dead plants, respectively. Finally, plants were uprooted at three months after inoculation to observe the roots and the internal discoloration in the rootstock. Wood sections from the stem (cultivar), and roots were collected for fungal reisolation that was conducted in Komada medium^6^. The frequency of isolation (%) was estimated as described before.

#### Infection using natural infested soil

Soil samples (*n* = 6) were collected throughout the affected area in the Portuguese almond orchard. Samples were collected from the rhizosphere of the plants at a depth no more than 30 cm. Prior to conduct the experiment, the number of propagules of the pathogen in the soil was estimated. To this end, a soil supension of each sample was prepared by adding 100 g of soil into to 250 ml Erlenmeyer flask with 100 ml of 0.05% water agar (Rokoagar AF LAB, ROKO Industrias, Llanera, Asturias, Spain), and homogeneized. For each soil sample, three replicated Erlenmeyer flask were used, and from each flask 1 ml of the supension was uniform distributed onto 9-mm Petri dishes filled with Komada medium^6^. Ten Petri dishes were plated per soil subsample, incubated for 48 h, washed under running tap water and incubated again until colonies developed. The number of *Fusarium*-like colonies of the ten replicated Komada Petri dishes per soil subsample was counted, with the propagule density being 116 CFUs per g of soil. The protocol was adapted from the soil dilution plate technique described by Leslie and Summerell^6^.

Almond plants of the cv. Lauranne grafted on both GF-677 and R20 rootstocks were transplanted in 2 L PVC pots filled with soil. As control, an additional lot of plants per each rootstock was transplanted in 2 L PVC pots filled with the same soil that was previously sterilized as described before for peat moss. There were six replicated plants per rootstock and type of soil (infested or sterilized soil), and they were arranged in a completely randomized design.

Plants were maintained in a greenhouse at 23 ± 2 °C under natural photoperiod over one year. Then, plants were uprooted to observe the roots and the internal discoloration in the rootstock. Wood sections were collected from the stem (cultivar), rootstock and roots for fungal reisolation which was carried out in Komada medium^6^. The frequency of isolation (%) was estimated as described before. In addition, the soil of each pot was removed and the final propagules density was estimated as described above.

#### Pathogenicity of * Fusarium* spp. strains from southern Portugal and Spain

The six *Fusarium* isolates indicated before were used in this experiment. Plants of the cv. Lauranne grafted on GF-677 rootstock were inoculated with the different isolates using conidial suspensions obtained from PDB as described before. Plants treated with only water were included as control. They were maintained in greenhouse conditions at 23 ± 2 °C for 3 months and irrigated three times per week. There were six replicated plants per fungal isolate or control, and they were arranged in a completely randomized design.

DS, incidence and mortality were evaluated as described before. At the end of the experiment, plants were uprooted to observe the roots and the internal discoloration in the rootstock. Wood sections were collected from the stem (cultivar), and roots (rootstock) for fungal reisolation which was carried out in Komada medium^6^. The frequency of isolation (%) was estimated as described before.

#### Data analysis

In all cases, data were tested for homogeneity of variances and normality prior to conduct ANOVA. For the first experiment, a factorial ANOVA was conducted with treatments (inoculated or control plants), rootstocks (GF-677 or R20), and their interaction as independent variables; and total length of shoots (cm), fresh weight of shoots (g), number of new sprouted buds, and total length (cm) and total fresh weight of new shoots (g) as dependent variables. For the second experiment, a factorial ANOVA was conducted with treatments (inoculated or control plants), rootstocks (GF-677 or R20), environmental conditions (optimal or stressed) and their interactions as independent variables and RAUDPC (%) as dependent variable. Data from the third experiment were not subjected to any statistical analysis since the main objective was to demonstrate the pathogenicity from natural soil infections in pots, and only symptoms observations were recorded. For the last experiment, a one-way ANOVA was conducted with fungal as independent variables and RAUDPC as dependent variable. In this later case, control data were excluded from the statistical analysis because no lesions developed. In all cases, means comparisons were performed using the protected Fisher’s LSD test at *P* = 0.05^44^. Data on the final incidence (% of affected plants) and mortality (% of dead plants) were analyzed by multiple comparisons for proportions tests at *P* = 0.05^48^. Data were analyzed using Statistix 10 software^45^.

### Research involving plants statement

This study was developed with commercial plants obtained from Spanish nurseries, therefore nonexotic or at risk of extinction, under controlled conditions, meeting all institutional, national and international guidelines and legislation for cultivated plants.

### Supplementary Information

Supplementary Table S1.

## Data Availability

All data generated and/or analyzed during this study are available from the corresponding author on reasonable request.
