# Supplementary material for: Identification and pathogenicity of Fusarium species associated with wilting and crown rot in almond (Prunus dulcis)
Source: Sci Rep. 2024 Mar 8;14:5720. doi: 10.1038/s41598-024-56350-5 (PMC10924081; doi:10.1038/s41598-024-56350-5)
Supplement: Supplementary file 1 — Supplementary Table S1. [file 41598_2024_56350_MOESM1_ESM.doc]

**Supplementary Table 1.** *Fusarium* spp. isolates and references sequences from GenBank used in the phylogenetic analysis.

| **Species** | **Isolate^x^** | **Host; Cultivar; rootstock; Scion** | **Origin** | **Special form** | **Collector; Date^y^** | **GenBank Accession no.^z^** | |
| --- | --- | --- | --- | --- | --- | --- | --- |
|  |  |  |  |  |  | ***tef1*** | ***rpb2*** |
| ***Fusarium oxysporum* species complex** |  |  |  |  |  |  |  |
| *F.callistephi* | **CBS 187.53^T^** | *Callistephus chinensis* | The Netherlands | *callistephi* | n/d | MH484966 | MH484875 |
| *F. carminascens* | **CBS 144739 = CPC 25792** | *Zea mays* | South Africa | n/d | C.M. Bezuidenhout | MH485025 | MH484934 |
|  | **CBS 144738 = CPC 25800^T^** | *Z. mays* | South Africa | n/d | C.M. Bezuidenhout | MH485028 | MH484937 |
| *F. contaminatum* | **CBS 111552** | Pasteurized fruit juice | The Netherlands | n/d | n/d | MH484991 | MH484900 |
|  | **CBS 117461** | Tetra pack with milky nutrition | The Netherlands | n/d | n/d | MH485002 | MH484911 |
| *F. cugenangense* | **CBS 620.72 = DSM 11271 = NRRL 36520** | *Crocus* sp. | Germany | *gladioli* | R. Schneider | MH484970 | MH484879 |
|  | **CBS 130304 = BBA 69050 = NRRL 25433** | *Gossypium barbadense* | China | *vasinfectum* | n/d | MH485012 | MH484921 |
| *F. curvatum* | **CBS 247.61 = BBA 8398 = DSM 62308 = NRRL 22545** | *Matthiola incana* | Germany | *matthiolae* | W. Gerlach | MH484967 | MH484876 |
|  | **CBS 238.94 = NRRL 26422 = PD 94/184T** | *Beaucarnia* sp. | The Netherlands | *meniscoideum* | J.W. Veenbaas-Rijks | MH484984 | MH484893 |
| *F. duoseptatum* | **CBS 102026 = NRRL 36115** | *Musa sapientum* cv. Pisang ambon | Malaysia | *cubense* | R.C. Ploetz | MH484987 | MH484896 |
| *F. elaeidis* | **CBS 217.49 = NRRL 36358** | *Elaeis* sp. | Zaire | *elaeidis* | T. Gogoi | MH484961 | MH484870 |
| *F. fabacearum* | **CBS 144742 = CPC 25801** | *Z. mays* | South Africa | n/d | C.M. Bezuidenhout | MH485029 | MH484938 |
| *F. glycines* | **CBS 176.33 = NRRL 36286** | *Linum usitatissium* | Unknown | *lini* | E.C. Stakman | MH484959 | MH484868 |
|  | **CBS 200.89** | *Ocimum basilicum* | Argentina | *basilici* | G. Tamiette | MH484979 | MH484888 |
| *F. gossypinum* | **CBS 116611** | *Gossypium hirsutum* | Ivory Coast | *vasinfectum* | K. Abo | MH484998 | MH484907 |
|  | **CBS 116613^T^** | *G. hirsutum* | Ivory Coast | *vasinfectum* | K. Abo | MH485000 | MH484909 |
| *F. hoodiae* | **CBS 132474^T^** | *Hoodia gordonii* | South Africa | *(hoodiae)* | O.A. Philippou | MH485020 | MH484929 |
| *F. languescens* | **CBS 645.78 = NRRL 36531^T^** | *Solanum lycopersicum* | Morocco | *lycopersici* | n/d | MH484971 | MH484880 |
|  | **CBS 302.91 = NRRL 36419** | *S. lycopersicum* | The Netherlands | *lycopersici* | D.H. Elgersma | MH484983 | MH484892 |
|  | **CBS 872.95 = NRRL 36570** | *S. lycopersicum* | n/d | *radicis-lycopersici* | n/d | MH484986 | MH484895 |
| *F. libertatis* | **CBS 144748 = CPC 25782** | *Aspalathus* sp. | South Africa | n/d | n/d | MH485023 | MH484932 |
| *F. nirenbergiae* | PV-827 | *P. dulcis* cv. Vairo/Garnem®; Rootstock | Íllora, Granada, Spain | n/d | A. Trapero & C. Agustí-Brisach; 09/30/2019 | ON493882 | ON407061 |
|  | PV-1046* | *P. dulcis* cv. Marinada/GF-677; Trunk | Toledo, Spain | n/d | B. I. Antón-Domínguez & C. Agustí-Brisach; 10/20/2020 | ON493883 | ON407062 |
|  | **CBS 129.24** | *Secale cereale* | Unknown | n/d | H.W. Wollenweber | MH484955 | MH484864 |
|  | **CBS 149.25 = NRRL 36261** | *Musa* sp. | Unknown | *cubense* | E.W. Mason | MH484956 | MH484865 |
|  | **CBS 181.32 = NRRL 36303** | *S. tuberosum* | USA | n/d | H.W. Wollenweber | MH484958 | MH484867 |
|  | **CBS 196.87 = NRRL 26219** | *Bouvardia longiflora* | Italy | *Bouvardiae* | B. Aloj | MH484977 | MH484886 |
|  | **CBS 127.81 = BBA 63924 = NRRL 36229** | *Chrysanthemum sp.* | USA | *chrysanthemi* | G.M. Armstrong | MH484974 | MH484883 |
|  | **CBS 130303** | *S. lycopersicum* | USA | *radicis-lycopersici* | J. Swezey | MH485014 | MH484923 |
|  | **CBS 744.79 = BBA 62355 = NRRL 22549** | *Passiflora edulis* | Brazil | *passiflorae* | W. Gerlach | MH484973 | MH484882 |
|  | **CBS 758.68 = NRRL 36546** | *S. lycopersicum* | The Netherlands | *lycopersici* | G. Weststeijn | MH484968 | MH484877 |
|  | **CBS 840.88^T^** | *Dianthus caryophyllus* | The Netherlands | *dianthi* | H. Rattink | MH484978 | MH484887 |
| *F. odoratissimum* | **CBS 794.70 = BBA 11103 = NRRL 22550** | *Albizzia julibrissin* | Iran | *perniciosum* | W. Gerlach | MH484969 | MH484878 |
|  | **CBS 102030** | *M. sapientum* cv. Pisang mas | Malaysia | *cubense* | R.C. Ploetz | MH484989 | MH484898 |
|  | **CBS 130310 = NRRL 25603** | *Musa* sp*.* | Australia | *cubense* | n/d | MH485013 | MH484922 |
| *F. oxysporum* | PV-452* | *P. dulcis* cv. Avijor/GF-677; Trunk | Monforte, Portugal | n/d | A. Trapero & C. Agustí-Brisach; 07/17/2017 | OR611986 | OR611989 |
|  | PV-453* | *P. dulcis* cv. Avijor/GF-677; Trunk | Monforte, Portugal | n/d | A. Trapero & C. Agustí-Brisach; 07/17/2017 | OR611987 | OR611990 |
|  | PV-534* | *P. dulcis* cv. Avijor/GF-677; Trunk | Monforte, Portugal | n/d | A. Trapero & C. Agustí-Brisach; 11/09/2017 | OR611988 | OR611991 |
|  | PV-548 | *P. dulcis* cv. Marta/GF-677; Trunk | Huelva, Spain | n/d | A. López-Moral & C. Agustí-Brisach; 02/04/2017 | ON493884 | ON4070623 |
|  | PV-571 | *P. dulcis* cv. Marta/GF-677; Trunk | Huelva, Spain | n/d | A. López-Moral & C. Agustí-Brisach; 02/04/2017 | ON493885 | ON407064 |
|  | **CBS 221.49 = IHEM 4508 = NRRL 22546** | *Camellia sinensis* | South East Asia | *medicaginis* | F. Bugnicourt | MH484963 | MH484872 |
|  | **CBS 144134^T^** | *S. tuberosum* | Germany | n/d | L. Lombard | MH485044 | MH484953 |
|  | **CBS 144135** | *S. tuberosum* | Germany | n/d | L. Lombard | MH485045 | MH484954 |
|  | **CPC 25822** | *Protea* sp. | South Africa | n/d | C.M. Bezuidenhout | MH485034 | MH484943 |
| *F. pharetrum* | **CBS 144750 = CPC 30822** | *Aliodendron dichotomum* | South Africa | n/d | F. van der Walt & G.J. Marais | MH485042 | MH484951 |
| *Fusarium* sp. A | PV-747* | *P. dulcis* cv. Lauranne/Garnem; Trunk | La Carlota, Córdoba, Spain | n/d | A. López-Moral & C. Agustí-Brisach; 09/12/2019 | ON493886 | ON407065 |
| *Fusarium* sp. A | PV-748 | *P. dulcis* cv. Lauranne®/Garnem®; Rootstock | La Carlota, Córdoba, Spain | n/d | A. López-Moral & C. Agustí-Brisach; 09/12/2019 | ON493887 | ON407066 |
| *Fusarium* sp. B | PV-804 | *P. dulcis* cv. Lauranne®/Garnem®; Rootstock | La Carlota, Córdoba, Spain | n/d | A. López-Moral & C. Agustí-Brisach; 09/12/2019 | ON493888 | ON407067 |
| *F. trachichlamydosporum* | **CBS 102028 = NRRL 36117** | *M. sapientum* cv. Pisang awak legor | Malaysia | *cubense* | R.C. Ploetz | MH484988 | MH484897 |
| *F. triseptatum* | **CBS 258.50 = NRRL 36389^T^** | *Ipomoea batatas* | USA | *batatas* | T.T. McClure | MH484964 | MH484873 |
|  | **CBS 116619** | *G. hirsutum* | Ivory Coast | *vasinfectum* | K. Abo | MH485001 | MH484910 |
| *F. veterinarium* | **CBS 109898 = NRRL 36153T** | Shark peritoneum | The Netherlands | n/d | C. Hoek | MH484990 | MH484899 |
|  | **NRRL 54984** | Mouse mucosa | USA | n/d | n/d | MH485036 | MH484945 |
| ***Fusarium fujikuroi* species complex** |  |  |  |  |  |  |  |
| *F. globosum* | **CBS 428.97^T^** | *Zea mays* seed | South Africa | n/d | n/d | MT010993 | MT010982 |
| *Fusarium mangiferae* | **NRRL 25226** | *Mangifera indica* | Israel | n/d | n/d | AF160281 | JX171622 |
| *F. proliferatum* | PV-787 | *P. dulcis* cv. Vairo/Garnem®; Trunk | Íllora, Granada, Spain | n/d | A. Trapero & C. Agustí-Brisach; 09/30/2019 | ON493889 | ON407068 |
|  | PV-814* | *P. dulcis* cv. Lauranne/Garnem®; Branch | La Carlota, Córdoba, Spain | n/d | A. López-Moral & C. Agustí-Brisach; 09/12/2019 | ON493890 | ON407069 |
|  | PV-825 | *P. dulcis* cv. Vairo/Garnem®; Trunk | Íllora, Granada, Spain | n/d | A. Trapero & C. Agustí-Brisach; 09/30/2019 | ON493891 | ON407070 |
|  | **GR_Fpb** | Asparagus | Spain | n/d | n/d | MT305198 | MT305140 |
|  | **GR_FP24** | Asparagus | Spain | n/d | n/d | MT305202 | MT305144 |
|  | **GR_FP172** | Asparagus | Spain | n/d | n/d | MT305208 | MT305150 |
|  | **SMFP3** | *Salvia miltiorrhiza* | China | n/d | n/d | MT371384 | MT934441 |
| *Fusarium udum* | **CBS 177.31** | *Digitaria eriantha* | South Africa | n/d | n/d | MH484957 | MH484866 |
| ***Fusarium redolens* species complex** |  |  |  |  |  |  |  |
| *F. redolens* | PV-600* | *P. dulcis* cv. Lauranne®/GF-677; Rootstock | Benamejí, Córdoba, Spain | n/d | B.I. Antón-Domínguez & C. Agustí-Brisach; 05/22/2018 | ON493892 | ON407071 |
|  | **GR-FR215** | Asparagus | Spain | n/d | n/d | MT305226 | MT305167 |
| *F. spartum* | **NRRL 66894** | *Macrochloa tenacissima* | Tunisia | n/d | n/d | MT409457 | MT409447 |
| ***Fusarium sambucinum* species complex** |  |  |  |  |  |  |  |
| *F. armeniacum* | **NRRL 6227** | Hay | n/d | n/d | n/d | HM744692 | HQ154480 |
| *F. langsethiae* | **NRRL 54940** | n/d | n/d | n/d | n/d | MW233138 | MW233482 |
| *F. sambucinum* | PV-572* | *P. dulcis* cv. Marta/GF677; Trunk | Huelva, Spain | n/d | A. López-Moral & C. Agustí-Brisach; 02/04/2017 | ON493893 | ON407072 |
|  | **FRC R4712** | n/d | n/d | n/d | n/d | MW233148 | MW233492 |
|  | **NRRL 31969** | n/d | n/d | n/d | n/d | MW233107 | MW233451 |
| *F. venenatum* | **FRC R-09186** | n/d | n/d | n/d | n/d | GQ915515 | GQ915499 |
|  | **MRC 2394** | n/d | n/d | n/d | n/d | MH582303 | MH582160 |
| *Dactylonectria estremocensis* | **CBS 129085^T^** | *Vitis vinifera* | Estremoz, Portugal | n/d | C. Rego & T. Nascimento. 2003 | JF735806 | KM232345 |

^x^Sequences from GenBank used in the phylogenetic analysis indicated in bold type. **T** = Ex-type isolates; **CBS**: Culture collection of the Centraalbureau voor Schimmelcultures, Fungal Biodiversity Centre, Utrecht, The Netherlands; **CPC**: Culture Collection of Pedro Crous; **NRRL**: Agricultural Research Service Culture Collection. Us Department of Agriculture, Mycotoxin Prevention and Applied Microbiology Research Unit, National Center for Agricultural Utilization Research, Agricultural Research Service, 1815 N University St., Peoria, IL 61604, USA; **PD**: Plant Protection Service, Wageningen, The Netherlands; **PV**: ‘Patología Vegetal’, Department of Agronomy, University of Cordoba, Spain;

^y^Collection date: month/day/year; n/d: non-determined.

^z^*tef1* = translation elongation factor 1-α; *rpb2* = RNA polymerase II subunit.

*Isolates selected for morphological characterization, study of the effect of temperature on mycelial growth, and pathogenicity tests.
